# Expanding narratives of governance constraints to improve coral reef conservation

**DOI:** 10.1111/cobi.13933

**Published:** 2022-07-11

**Authors:** Rachel A. Turner, Johanna Forster, Clare Fitzsimmons, Robin Mahon

**Affiliations:** ^1^ Environment and Sustainability Institute University of Exeter Penryn UK; ^2^ School of International Development University of East Anglia Norwich UK; ^3^ Tyndall Centre for Climate Change Research University of East Anglia Norwich UK; ^4^ School of Natural & Environmental Sciences Newcastle University Newcastle upon Tyne UK; ^5^ Centre for Resource Management and Environmental Studies University of the West Indies Cave Hill Campus Barbados

**Keywords:** Caribbean, environmental governance, governability, interactive governance, perceptions, Caribe, gobernanza ambiental, gobernanza interactiva, gobernabilidad, percepciones, 交互式治理, 管理能力, 观念, 加勒比地区

## Abstract

To understand and address the failures of reef governance, it is critical to understand the perceptions of diverse policy makers and practitioners about the challenges they face in achieving their goals. Examining the discourse of policy makers and practitioners can reveal the extent to which these perceptions capture the full spectrum of potential governance challenges, including those related to management, institutional structures and processes, the values and principles underpinning governance, and the social and environmental context. We conducted semistructured interviews with 110 policy makers and practitioners across multiple sectors, scales, and contexts in Barbados, St Kitts and Nevis, Belize, and Honduras. We used thematic qualitative analysis informed by theories of interactive governance and governability to examine the challenges perceived by governance actors. Perceived governance challenges were broadly consistent across countries, but differed by sector (*V* = 0.819, *F*_6,60_ = 1.502, *p* = 0.01) and by level (community compared with national) (*V* = 0.194, *F*_1,10_ = 2.178, = 0.026). Management inputs and outputs, challenges relating to the socioeconomic context, issues of leadership and power, and stakeholder engagement were commonly cited challenges (>75%). Few respondents discussed challenges relating to the ecological context, governance processes, or the values and principles underpinning governance. We argue that examining perceptions can inform efforts to improve governance and assess the appropriateness of particular management tools under context‐specific governance constraints. Furthermore, expanding the narratives of governance challenges to encompass the subtle values and images underpinning governance, and the scale of the challenges faced, can help identify a wider set of opportunities for change.

## Abstract (es)

Expansión de las Narrativas de los Límites de la Gobernanza para Mejorar la Conservación de los Arrecifes de Coral

**Resumen:**

Es muy importante entender las percepciones que tienen los practicantes y los formuladores de políticas sobre los retos que enfrentan para alcanzar sus objetivos para poder entender y abordar los fracasos en la gobernanza de los arrecifes. El análisis del discurso de los formuladores y los practicantes puede revelar la extensión a la que estas percepciones capturan el espectro completo de los retos potenciales para la gobernanza, incluidos aquellos relacionados con el manejo, las estructuras y los procesos institucionales, los valores y principios que apuntalan la gobernanza y el contexto social y ambiental. Realizamos entrevistas semiestructuradas a 110 formuladores y practicantes de múltiples sectores, escalas y contextos en Barbados, San Cristóbal y Nieves, Belice y Honduras. Usamos un análisis cualitativo temático informado por las teorías de la gobernanza interactiva y la gobernabilidad para examinar los retos percibidos por los actores de gobernanza. A grandes rasgos, los retos percibidos en la gobernanza fueron coherentes entre los países, pero difirieron por sector (*V* = 0.819, *F*_6,60_ = 1.502, *p* = 0.01) y por nivel (comunitario comparado con nacional) (*V* = 0.194, *F*_1,10_ = 2.178, = 0.026). Las aportaciones y producciones del manejo, los retos relacionados con el contexto socioeconómico, los temas de liderazgo y poder y la participación de los actores fueron los retos mencionados comúnmente (>75%). Pocos respondientes discutieron los retos relacionados con el contexto ecológico, los procesos de gobernanza o los valores y principios que apuntalan la gobernanza. Alegamos que el análisis de las percepciones puede guiar a los esfuerzos para mejorar la gobernanza y evaluar cuán apropiadas son las herramientas particulares de manejo bajo los límites de gobernanza específicos al contexto. Además, expandir las narrativas de los retos de gobernanza para englobar los valores e imágenes sutiles que apuntalan la gobernanza, y la escala del reto al que se enfrenta, puede ayudar a identificar un conjunto más amplio de oportunidades de cambio.

## 【摘要】

为了理解和解决珊瑚礁管理的失败, 需了解不同的政策制定者和实践者对实现目标时所面临挑战的看法。研究决策者和实践者的话语可以揭示这些看法在多大程度上反映了潜在的管理挑战, 包括与管理、制度结构和程序、支持管理的价值和原则, 以及社会和环境背景有关的挑战。我们对巴巴多斯、圣基茨和尼维斯、伯利兹和洪都拉斯涉及多个部门、规模和背景的110名决策者和实践者进行了半结构化访谈。我们以交互性管理和管理能力理论为指导, 使用主题定性分析, 研究了管理者所感知到的挑战。各国受访者感知到的管理挑战大体一致, 但因部门(*V* = 0.819, *F*_6,60_ = 1.502, *p* = 0.01)和级别(社区或国家, *V* = 0.194, *F*_1,10_ = 2.178, *p* = 0.026)而有所不同。普遍提到的挑战(>75%)包括管理投入和产出、与社会经济环境有关的挑战、领导力和权力问题, 以及利益相关者的参与。很少有受访者讨论与生态环境、管理过程或支持管理的价值观与原则有关的挑战。我们认为, 对观念的研究可以为改进管理提供信息, 还可以评估特定背景的管理制约因素下管理工具的适当性。此外, 扩大对管理挑战的叙述, 以包括支持管理的不易察觉的价值观和形象, 以及所面临的挑战的规模, 可以帮助确定更广泛的变革机会。**【翻译:胡怡思;审校:聂永刚】**

## INTRODUCTION

The plight of coral reefs globally is a long‐standing concern in environmental science and conservation research. The ecological state of coral reefs has deteriorated rapidly.

Global coverage of living coral and the capacity of coral reefs to provide important ecosystem services to coastal communities have declined by half since the 1950s (Eddy et al., 2021). The diverse threats coral reefs face and the failures to halt their decline are often attributed ultimately to poor governance (Christie & White, 2007; Forster et al., 2017; Hughes et al., 2017b; Sale, 2008), leading to the widespread calls for an overhaul of governance arrangements, especially in light of rapid and complex social and environmental changes that present new challenges and intensifying pressures (Hughes et al., 2017a; Morrison et al., 2020a; Andrello et al., 2021).

Despite calls for governance reform, literature on the effectiveness of coral reef conservation is dominated by a relatively narrow focus on developing and evaluating management measures that aim to reduce human pressures, such as protected areas or fisheries management tools. Mounting evidence suggests that this focus has led to dominant conservation tools being widely advocated, often without sufficient consideration of the capacity of the governance system to implement them (Chuenpagdee & Jentoft, 2007; Chuenpagdee, 2011). This has resulted in protected areas that lack effective governance and management and that consequently provide few conservation benefits (Agardy et al., 2011; Jentoft et al., 2007). Similarly, comanagement approaches to conservation are widely promoted but can be undermined by a failure to consider communities’ willingness and capacity to engage in resource governance (Gelcich et al., 2009; McConney & Pena, 2012) or to adequately engage with issues of environmental social justice (Gurney et al., 2021). The potential conservation benefits of coral reef management tools can, therefore, be undermined by overoptimism about governance capacity and a lack of knowledge about context‐specific governance challenges.

Furthermore, a focus on management measures arguably gives insufficient attention to the underpinning governance structures and processes, questions of power and agency, and the values and worldviews that shape governance goals and outcomes. A substantial body of research emerging from common pool resource management theory has directed attention to the structural characteristics of governance systems that promote effective natural resource management (Ostrom, 2009; Cox et al., 2010). A diagnostic approach has been used to examine how different combinations of institutional design features are associated with positive or negative outcomes (Ostrom, 2007; Cinner et al., 2012; Basurto et al., 2013). More qualitative perspectives on marine governance have contributed in‐depth case studies that highlight how power dynamics, conflict, agenda setting, and processes of inclusion and exclusion influence the ways in which governance systems evolve to pursue particular goals (Chuenpagdee et al., 2013b; Scholtens, 2015; Blythe et al., 2017; Morrison et al., 2019a). Such processes often present challenges that can undermine effective governance, for example, through competing priorities or resisting changes to the status quo (Fortnam, 2019). Underlying these challenges are deep‐held values, images (of the nature of governance systems and the problems they seek to address), and principles, which can differ among stakeholders and are slow to change (Song et al., 2013). The extent to which some of these less tangible aspects of governance are perceived as constraints by policy makers and practitioners in comparison with more practical challenges of management is seldom explored.

We argue that to understand and address the failures of coral reef governance, it is critical to examine how actors in reef governance systems perceive the governance challenges or constraints that they face. The importance of understanding these perceptions is two‐fold. First, understanding the perceptions of diverse policy makers and practitioners across multiple scales and sectors is essential to identify the real‐world challenges they face in achieving their goals. Governance systems for coral reefs involve diverse stakeholders, and complex cross‐scale and cross‐sectoral dynamics mean there is a need to understand a range of perspectives on where governance challenges lie. This includes an understanding of the perceived effectiveness of management tools and challenges to their implementation; the ways in which governance structures and processes enable or hinder effective management; and the question of what is feasible in a given social and environmental context. Identifying common and diverging views of governance capacity across different contexts can inform efforts to strengthen coral reef governance. Where governance constraints are difficult to overcome, knowledge of these constraints can also inform the selection of management measures that may be better suited to particular contexts.

Second, examining these perceptions can indicate whether the expanding dialogue on reef governance––from management tools to wider governance structures and processes––is reflected in the narratives of practitioners and policy makers who are actively shaping governance systems on the ground. Effective governance reforms that support transitions to more positive social and environmental outcomes require attention to all aspects of governance and addressing gaps in the discourse on governance challenges could help identify a wider set of opportunities for change. Though academic theory around natural resource governance has shifted to include a wider range of considerations, this must be mirrored in understanding and action by governance actors in order to create change (Ziegler et al., 2019). Shared understanding of mental models and narratives of the nature and causes of environmental problems can help avoid conflict when identifying solutions (Brewer, 2013; Song et al., 2013).

We examined the perceptions of policy makers and practitioners engaged in coral reef governance in four countries of the Wider Caribbean Region. We investigated the range of challenges perceived by actors in reef governance systems; extent to which they vary by country, level of governance, or sector; and extent to which the discourse of policy makers and practitioners reflects the breadth of the scientific discourse as described above, considering challenges related to management, institutional structures and processes, and values and principles that underpin governance.

## METHODS

### Study area

The Wider Caribbean Region provides a unique context to investigate coral reef governance. Caribbean coral reefs hold exceptionally high biodiversity but have experienced rapid ecological decline (Hoegh‐Guldberg et al., 2007; Jackson et al., 2014). They are threatened by growing demand for marine resources and impacts from climate change (Mora, 2008). Recognition of these threats and the need for cross‐scale solutions has led to calls for improved multilevel governance structures (Mumby & Steneck, 2008; Mahon et al., 2014). However, geopolitical diversity, complex jurisdictions, and overlapping responsibilities in the region present challenges to effective governance at national and regional scales (Fanning et al., 2007, 2009). Four study countries were selected to reflect some of this diversity, spanning both island and continental nations with varying extents of coral reef habitats, threats, and histories of conservation: Barbados, St Kitts and Nevis, Belize, and Honduras (Bay Islands). In each country, three coastal communities and national‐level governance actors were studied to capture a diversity of stakeholders and resource uses.

Governance systems differ across the four study countries. In the island nations of Barbados and St Kitts and Nevis, of which the latter is a federation, national government departments are the main actors in coral reef governance, and there is little distinction between national and local governance aside from the island‐level administration for Nevis. Few local organizations are involved in reef governance. In contrast, in the two continental states of Belize and Honduras, both local government and nongovernmental organizations (NGOs) play a greater role. In Belize, this includes legally mandated town councils or informal village councils. In Honduras, municipal government departments have some responsibility for decision‐making, implementation, and enforcement. More complex governance structures in the continental nations, together with a longer history of marine conservation, mean that a wider range of actors are incorporated in governance processes, including through comanagement arrangements with local NGOs (Cho, 2005; McConney et al., 2007). Marine resource users are well organized in Belize through cooperatives and associations, but less so in Honduras and the island nations.

The social and environmental context of coral reefs and their use differs across study countries. Barbados and St Kitts and Nevis have narrow shelf areas and a smaller extent of coral reefs compared with Belize and Honduras, both of which have associated coastal islands and include part of the large Meso‐American Barrier Reef System (MBRS). Reefs around Barbados and St Kitts and Nevis are considered threatened by human activities, including overfishing and coastal development (Burke et al., 2011). Sediment and pollution from land‐based sources and coastal development are considered major threats to reef health in Belize and Honduras, and overfishing is a significant threat to reefs in the MBRS as a whole (Burke & Maidens, 2004). Across all countries, rising sea temperatures, coral bleaching, and increasing intensity of hurricanes and storms have long been recognized to exacerbate reef decline (Wilkinson & Souter, 2008; Agostini et al., 2010).

The study countries reflected a range of development, with higher poverty levels in Belize and Honduras (41% and 65% below the poverty line, respectively) (CIA, 2013). Tourism is the primary source of foreign exchange in Barbados, Belize, and St Kitts and Nevis; many activities focus on the reef and nearshore areas. In Honduras, rapid growth of tourism in the Bay Islands has increased stressors on coral reefs through unregulated development (Moreno 2005 et al., 2005; Harborne et al., 2011). Fisheries contribute less than tourism to national economies but play an important role in all four countries. In Barbados, reef fishes form a relatively small component of landings, although the fishing industry is considered a social safety net (McConney et al., 2003). High local demand for reef fishes and the importance of marine exports has led to overexploited nearshore fisheries in St Kitts and Nevis (CRFM, 2011). In Belize and Honduras, fisheries are important for local consumption and exports, including high‐value species, such as lobster and conch. Small‐scale fisheries in Belize are concentrated in shallow waters of the barrier reef and atolls. Close proximity to the continental shelf edge in Honduras allows small‐scale fishers to target reef‐related and pelagic species (Box & Canty, 2010).

### Data collection

Semistructured interviews were conducted with 110 governance actors comprising individuals involved in reef management, decision‐making, or policy in each country at local and national levels (Table 1). Although we recognize the important, direct role played by resource users in local governance systems, it was beyond the scope of this work to capture these perspectives comprehensively (see Turner et al. [2014, 2017] for analyses of resource‐user perceptions of coral reef governance). We included local resource users only where they acted in a representative capacity in wider decision‐making processes, for example, as leader of a local fisheries cooperative or tour operator association. Interviewees operating in the three case study communities in each country were classified as local, whereas those with a broader remit were classified as national. Respondents were selected based on preliminary searches and subsequent snowball sampling, selecting respondents purposively to represent the broad range of reef governance actors. Respondents worked across a range of sectors and in several types of organizations, spanning government departments, NGOs, industry bodies, and educational institutions. Sample sizes reflect the varying complexity of governance arrangements across the study sites. Although perceptions of governance challenges may be influenced by individual knowledge and experience, interviewees represented a range of experience in each country. Interviews were conducted between February 2011 and August 2012, lasted 45–90 min, and were recorded and transcribed where permitted. Interviews included open‐ended questions about a range of topics (Appendix S1). Specific questions designed to elicit perceptions of governance challenges asked respondents about management activities they would like to pursue but felt unable to and more generally whether they perceived challenges to managing reefs effectively. The entire interview was included in the analyses because respondents frequently referred to governance challenges when responding to open‐ended questions.

**TABLE 1:** Summary of sample of 110 governance actors participating in semistructured interviews

Respondent type		Barbados	St Kitts and Nevis	Honduras	Belize	Total
Level	local	5	1	20	23	49
	national	9	24	13	15	61
Sector	community	0	0	1	2	3
	conservation	3	4	12	15	34
	enforcement	1	2	6	2	11
	environment	2	5	7	3	17
	fisheries	3	8	5	5	20
	research	2	2	0	1	6
	tourism	3	4	2	10	19
Total		14	25	33	38	110

### Data analyses

Transcripts were coded in NVivo 9 by two researchers, with cross‐checking to ensure consistency in code development and interpretation. First, inductive coding identified the different constraints that respondents perceived to managing coral reefs effectively. Second, these constraints were grouped into themes, informed by the theoretical framework of interactive governance (Kooiman et al., 2005). The interactive governance approach offers a useful lens to examine narratives of governance challenges because it draws attention to three orders of governance that provided an analytical framework to differentiate challenges relating to management activities, institutional structures and processes, and the values and principles that underpin governance, enabling analysis of the extent to which each of these are represented in the narratives of reef governance actors. The first order involves problem identification and formulation of solutions, encompassing day‐to‐day decision‐making. The second order relates to the design of appropriate institutions (such as norms, laws, and organizations) and instruments (such as regulations, incentives, and procedures) to solve problems or create opportunities. The third order, or meta‐order, involves the deliberation of values and principles that shape the goals of governance and underpin the roles of governing actors. Linked to the interactive governance framework is the concept of governability, which examines governance capacity by considering the properties of the governing system and the system to be governed (Jentoft, 2007; Chuenpagdee et al., 2013a). The system to be governed includes the ecological and social components of natural resource systems, which can be diverse, complex, and dynamic, presenting inherent challenges to those who seek to govern it. We used the three orders of governance and the social and ecological context of governance as a framework to examine the governance challenges identified (Table 2).

**TABLE 2:** Coding scheme used in analysis of semistructured interview data showing the orders of governance and the corresponding components of governance systems, key themes within each order, number of inductively generated codes mapped on to each theme, and frequency that each theme was mentioned

					Frequency mentioned (%)				
Governance order	Governance component	Theme	Code	Description of content coded under theme	BBD (*n* = 14)	SKN (*n* = 25)	BZE (*n* = 38)	HON (*n* = 33)	Total (*n* = 110)
Context	system to be governed	socioeconomic contexts	29	general and specific issues relating to socioeconomic pressures influencing reef governance, including poverty and livelihood dependency, economic and cultural context, and social problems (e.g., drugs and crime)	93	92	92	88	91
		ecological context	7	attributes of natural systems influencing reef management, including complexity and issues of scale (e.g., drivers of change, ecological connectivity, and temporal dynamics)	43	36	55	36	44
First order	management	inputs	3	resources and capacity for management, changes in funding climate, and reliance on project funding	79	88	89	91	88
		processes	5	misuse and ineffective use of resources, issues relating to the nature of management activity (e.g., ad hoc and reactive)	29	40	29	39	35
		outputs	14	general and specific issues relating to the implementation of reef management activities and their enforcement	100	100	97	100	99
Second order	governing system	institutional structures	5	general and specific issues relating to the institutional arrangements in place for reef governance, including issues relating to scale and clarity of roles and responsibilities	64	52	58	55	56
		leadership and power	13	issues of political commitment and prioritization, level of authority and leadership, and issues relating to conflicts of interest, corruption, and susceptibility to public pressure	93	100	84	91	91
		legislation and regulations	6	general and specific issues relating to weak or absent policy, legislation and regulation, role of informal governance and historical legacy, and mechanisms for change	79	84	68	73	75
	governance interactions	engagement and participation	9	issues relating to stakeholder engagement, support for management, collective action, stewardship, and voice	93	88	82	85	85
		research and information	6	availability, coordination, and dissemination of scientific research and information, uptake in decision‐making	79	60	58	70	65
		connectivity	3	level of cooperation and integration among governing bodies	79	60	74	82	74
Third order	governing system	metagovernance	5	clarity of goals for reef governance, underlying values and culture, issues relating to lack of common and long‐term vision	57	40	24	36	35
	governance interactions	process quality	7	issues relating to the legitimacy of reef governance, fairness, accountability, transparency, flexibility, and trust	71	56	63	76	66

Abbreviations: BBD, Barbados; BZE, Belize; HON, Honduras; SKN, St Kitts and Nevis.

In total, 112 individual codes were generated (Appendix S2), each of which was mentioned by 1–102 respondents and on average by 19 respondents. These codes were categorized under 13 themes corresponding to different aspects of the governance framework (Table 2). We present a quantitative overview of the data, summarizing the frequencies of responses across themes in relation to study sites, governance levels, and the different aspects of governance. These quantitative indicators of governance quality can support monitoring, aid initial diagnosis of governance weaknesses, and enable some generalization across contexts (Kaufmann et al., 2000; Engle & Lemos, 2010). Coding matrix queries in NVivo were used to identify the number of respondents referring to each theme. Multivariate analysis of variance was conducted to explore whether perceptions of governance themes differed significantly among respondents in different locations (country and level) and roles (sector).

## RESULTS

The number of codes generated in relation to each theme varied (Table 2). The largest number of codes related to perceived challenges in the social and economic context within which governance was taking place; challenges relating to management outputs; and issues of leadership and power. The number of codes associated with each theme may reflect the specificity of some of the challenges discussed compared with others. For example, some themes, such as socioeconomic context, generated a large number of codes, reflecting distinct local challenges; others, such as issues of connectivity, were discussed in more general terms. The number of codes generated under each theme may also indicate the extent to which each of the governance components were considered by the respondents and, therefore, the breadth of issues discussed.

### Perceptions of governance challenges

Overall, each theme was mentioned by 35–100% of respondents (Table 2). Though many of the themes incorporated a diverse range of specific issues (Appendix S2), there was a broadly common perception of the challenges to coral reef governance; 10 of the 13 themes were mentioned by over 50% of respondents. The most commonly mentioned themes broadly corresponded to those that generated greater numbers of codes. Management outputs, including implementation and enforcement of specific management actions, were mentioned by 99–100% of respondents across all countries. Challenges relating to the socioeconomic context, management inputs, leadership and power, and issues of engagement and participation were also mentioned by over 75% of respondents in each country. In contrast, fewer respondents discussed challenges relating to the ecological context, management processes, and topics relating to metagovernance.

### Differences in perceptions of challenges

The frequency with which governance themes were perceived did not differ significantly across the study countries (*V* = 0.300, *F*_3,30_ = 1.022, *p* = 0.438). There were, however, qualitative differences within themes that highlighted the context‐specific challenges faced by respondents in different settings, shown in the varying frequency that individual codes were discussed within each theme (Appendix S2). These differences reflected variation in the social and environmental contexts, including, for example, higher levels of poverty and livelihood dependence on reefs in Belize and Honduras and differences in the types of management tools implemented. For example, marine protected areas are common in Belize and Honduras but not in St Kitts and Nevis or Barbados.

**FIGURE 1:**
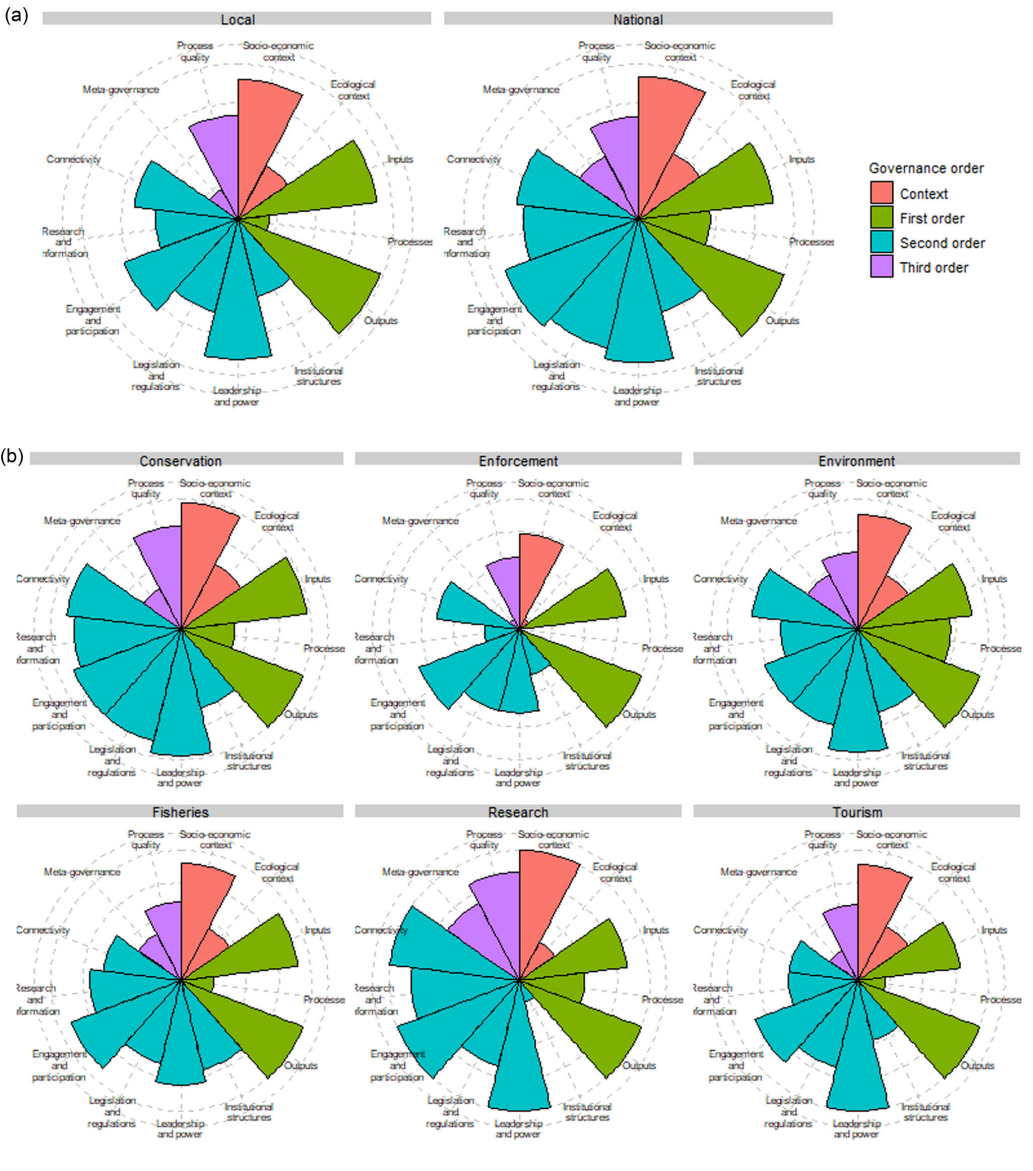
Percentage of respondents perceiving challenges coded under each governance theme for (a) local (*n* = 49) and national (*n* = 61) actors and (b) conservation (*n* = 34), enforcement (*n* = 11), environmental (*n* = 17), fisheries (*n* = 20), research (*n* = 6), and tourism (*n* = 19) sectors. Respondents categorized under the community sector are not included because of the small sample size (*n* = 3)

Local‐level respondents had different perceptions of governance challenges to national‐level respondents (*V* = 0.194, *F*_1,10_ = 2.178, *p* = 0.026) (Figure 1a). In particular, local actors were less concerned with management processes (perceived by 20% local compared with 46% national); legislation and regulations (61% compared with 85%); research and information (53% compared with 74%); and metagovernance (22% compared with 46%). In contrast, local actors’ concerns about the socioeconomic context of reef governance, management inputs and outputs, and leadership and power were similar to those of national actors (Figure 1a).

Perceptions of governance challenges also differed by sector (*V* = 0.819, *F*_6,60_ = 1.502, *p* = 0.01) Figure (1b). Some constraints were commonly perceived across all sectors, including challenges relating to the socioeconomic context, management inputs and outputs, and engagement and participation (all perceived by >70% of respondents in each sector). Issues of leadership and power and legislation and regulations were also commonly discussed across all sectors (>60%). Respondents from the conservation, fisheries, and environment sectors more commonly discussed constraints relating to the ecological context and institutional structures. Issues around metagovernance were most commonly discussed by respondents in the research sector (67%) compared with all other sectors (below 50%). Researchers and those in the conservation sector also more commonly mentioned issues relating to research and information, connectivity, and quality of governance processes. Overall, those in the enforcement sector noted fewer governance challenges, perceiving 7 of the 13 themes least frequently.

## DISCUSSION

Our findings suggest that actors in coral reef governance systems perceive a range of common governance challenges. Though we did not explicitly explore the relative importance of these, we assumed that commonly discussed issues reflect challenges respondents perceived to be important. There was little difference in the themes discussed across the four study countries, despite these encompassing diverse social, economic, cultural, political, and ecological systems. The underlying coding showed that the specific nature of the challenges within each theme differed across sites in some cases, whereas in others, they comprised a similar set of common constraints.

It is more difficult to interpret why some themes were not commonly discussed. We suggest three possible explanations. First, the themes may not have represented a constraint or problem to the respondents (in some cases because they have been considered outside the remit of their role). Second, respondents may not have been aware of the issues. Third, the themes may have been alluded to but not discussed directly and were thus underrepresented in data coding. Despite these challenges of interpretation, these data provide a useful indication of how policy makers and practitioners perceive governance and the challenges they face, informing a greater understanding of their perspective on what makes a system more or less governable.

### Key challenges perceived

First‐order challenges related to management inputs and outputs were nearly ubiquitous among respondents, indicating a clear focus on these governance issues. Despite emphasis on the evaluation and refinement of coral reef management tools in academic and gray literatures, their implementation and enforcement remain a challenge in many contexts. It is well established that capacity shortfalls constrain the effectiveness of management tools, with limited human and financial capacity among the most important factors explaining ecological outcomes of marine protected areas (MPAs), for example (Gill et al., 2017).

Several second‐order governance challenges were also commonly mentioned. Challenges perceived in relation to leadership and power often reflected constraints of working within hierarchical governance structures, where limited higher‐level prioritization and a lack of authority at local scales make governance difficult. Hierarchical reef governance systems and top‐down regulations inherited from colonial administrations often prove ineffectual for resource management and conservation because monitoring and enforcement are challenging, where resource use is rural and dispersed (Mahon, 2008). Correspondingly, challenges of community engagement were commonly mentioned, particularly by national‐level respondents. Although Honduras and Belize have experienced recent transitions toward comanagement, this can prove challenging because of inadequacies in (often small) government departments, as well as the limited capacity of resource user organizations (Mahon, 2008; McConney & Pena, 2012). This reflects a wider problem of policy layering in which new approaches, such as comanagement, are applied without sufficient attention to existing governance weaknesses, often leading to substantial implementation challenges (Kelly et al., 2019). Moves toward greater sharing or devolution of power can also engender resistance in defense of the status quo (Fortnam, 2019), and power imbalances can pose a challenge to governability (Chuenpagdee & Jentoft, 2009). Consequently, though challenges discussed reflected the differing governance structures in place, themes of power and leadership were prevalent across all countries. Addressing the underlying weaknesses of existing governance systems to support more effective collaborative governance may require an enabling approach that promotes self‐organization, local cooperation, and effective resource user organizations (Mahon, 2008). It may also require continued improvements to broader governance systems to shift power away from actors, such as industry lobbies, that can influence government priorities (Morrison et al., 2020b).

Second‐order issues of connectivity and institutional structures reflected concerns about the sectoral nature of coral reef governance, commonly discussed by respondents from the conservation, fisheries, and environment sectors. Given the complex set of drivers influencing reef health and the diversity of stakeholders involved (Forster et al., 2017), there is no single authority responsible for coral reef governance, leading to a lack of clarity around roles and responsibilities, and challenges of connectivity and information‐sharing across sectors. Although institutional diversity can have benefits for addressing complex challenges (Baird et al., 2019), fragmentation can be a problem (Kelly et al., 2019). Reframing narratives about coral reef conservation to highlight interconnected goals and mutual interests may support more integrated approaches to reef governance, while also supporting the achievement of other biodiversity and sustainable development goals (Morrison et al., 2019b).

Finally, commonly discussed challenges related to the socioeconomic context have implications for understanding governability. Ability to govern is determined not only by the capacity of the governing system, but also by the characteristics of the system to be governed, including aspects of social systems, such as stakeholder diversity, level of conflict, and mobility (Kooiman et al., 2008). The prevalence of this theme across all countries and sectors reflects that human pressures are an important challenge for achieving effective resource governance and remain difficult to integrate in decision‐making. Recent research suggests that the potential for local management to contribute to environmental goals is strongly linked to the level of human pressure (Cinner et al., 2020). High human pressure exerts great anthropogenic influence on coral reefs, and high dependence of coastal populations on natural resources can lead to potential conflicts and trade‐offs between social and environmental objectives of reef governance. These challenges may indicate a poor fit between the images of how a system should be governed (and the management tools associated with these) and the diversity and complexity of local contexts (Mahon, 2008). For example, marine protected areas are often not well supported in contexts of high resource dependence. This was evident in St Kitts and Nevis, where despite top‐down authority for coral reef governance, there was clear political reluctance to impose restrictions on fisheries. Correspondingly, concerns about the fairness of governance processes and their outcomes were also common, reflecting contemporary debates about equity and environmental social justice in conservation (Dawson et al., 2018; Friedman et al., 2018). Compounding these challenges, coastal management agencies often have limited skills in social science, and attention to livelihoods, though increasing, often fails to provide viable alternative or supplementary income (McConney & Pena, 2012). Governability may be enhanced by the evaluation of and attunement to the social context (Bavinck et al., 2008).

### Gaps in the discourse

Metagovernance, or third‐order governance, was the least commonly mentioned theme, capturing more intangible issues, such as the clarity of goals for reef governance, underlying values, and challenges of establishing a shared long‐term vision. The low occurrence of this theme may indicate that these issues are not perceived to present a challenge for effective coral reef governance or that the subtle nature of these challenges may lead to them being underrepresented in the data coding. Arguably though, other explanations for the limited discussion of these issues are plausible. Given the focus of the interviews on coral reef governance, respondents may not have made a conceptual connection between resource management and lofty topics, such as principles and values, that are deeply ingrained (Song et al., 2013) and may be taken as given and not up for discussion. If there are few opportunities for debate about the goals of governance, respondents may have focused on discussing the challenges they perceive within the confines of the status quo. Similarly, with respect to management, discussion centered on inputs and outputs; little attention was paid to management processes. Issues around metagovernance were most commonly discussed by respondents in the research sector, reflecting the more abstract nature of these ideas and the role of researchers as having an outsider perspective, typically less embedded in the day‐to‐day processes of coral reef governance. Greater awareness among researchers also reflected that academic governance literature is often very theoretical and can be inaccessible to policy makers and practitioners (Bennett & Satterfield, 2018). In addition, local actors discussed these issues less often than national actors, which may correspond to the nature of hierarchical systems, where local governors focus on first‐order implementation and problem solving, whereas agenda‐setting tends to take place at higher levels. Even at a national level, this may predominantly entail signing up to the principles and values embedded in multilateral environmental agreements, which may not be translated into local action or taken up by communities (Mahon, 2008). If leveraged effectively by informed local actors, though, international efforts toward more integrated and scaled‐up approaches (e.g., those of the UNESCO World Heritage Centre and Ramsar Convention on Wetlands of International Importance) can support the reframing of national goals and priorities for reef governance (Morrison et al., 2020b; Bridgewater & Kim, 2021).

Surprisingly, given the strong focus on the social context of reef governance, there was little discussion of challenges related to the ecological context. Challenges of diversity, complexity, dynamic and nonlinear change, and interconnectedness and scale in managing ecological systems are well documented in the literature, reflecting characteristics of the system to be governed that can limit governability (Folke et al., 2007; Chuenpagdee & Jentoft, 2009; Berkes, 2010). The application of particular management tools also requires attention to the wider environmental context because tools, such as MPAs, may be less effective where wider seascapes are degraded (Cinner et al., 2020). The low occurrence in interviews of challenges related to the environmental system may reflect a perception that they are beyond the ability of local governance actors to control. Research on recreational fisheries social‐ecological systems similarly shows that stakeholders have lower awareness of the wider environment and governance system compared with the attributes of the resource system and influence of actors (Ziegler et al., 2019). The authors suggest that respondents might have viewed these slow‐moving variables as “fixed contextual settings” (Ziegler et al., 2019: 1043). These issues were discussed more often by respondents in conservation, fisheries, and environment sectors, perhaps reflecting heightened awareness of these challenges in comparison with tourism or enforcement sectors.

The ecological context theme included codes capturing challenges related to the scale of drivers influencing reef heath, yet respondents discussed this topic infrequently. Mahon (2008) suggests that Caribbean reefs are typically not treated as transboundary systems, despite ecological connectivity across borders (e.g., via larval dispersal). Underlying coding showed that some issues of scale were more commonly discussed in Belize, perhaps reflecting the country's significant responsibility in relation to the transboundary MBRS. More broadly, our findings are in stark contrast to the increasing recognition that the escalating impacts of global climate change present some of the greatest threats to reef health, with calls to radically reframe the problem of coral reef governance to focus on these distal drivers and the actors responsible for them (Morrison et al., 2020a). Consideration of institutional fit to the scale of environmental (and social) problems is important for effective governance; mismatches of spatial scale are a common reason for management failure (Berkes, 2010; Epstein et al., 2015). Institutional fit can be enhanced through the presence of cross‐scale linkages (Fanning et al., 2013), a challenge that was more commonly discussed by respondents under the theme of connectivity.

### Implications

In calls for governance reform in coral reef conservation, the term *governance* is often used loosely and the changes required lack the specificity to permit effective implementation. We have examined the narratives of policy makers and practitioners, whose perspectives shape the reality of evolving coral reef governance on the ground. The perceptions of practitioners are not static and are likely to change over time in response to changing circumstances. For example, since these data were collected, the acceleration of the climate crisis and its impact on coral reefs could mean that climatic and other large‐scale ecological change may now feature more strongly in local governance discussions. Nevertheless, these data remain highly relevant, recent literature confirming that the pressing concerns of first‐order governance issues continue to be prominent challenges. There is little indication of a transition toward greater reflexivity around the goals and values underpinning coral reef governance at local and national scales.

Examining perceived governance constraints can inform an understanding of what makes a system more or less governable, providing a foundation for improvement. Acknowledging the common challenges perceived can inform an assessment of governability that considers conflicts, vested interests, and power struggles, as well as the more tangible concerns of limited resources and capacity and high dependence on coral reefs. Deliberate action to improve governance may be taken through adjustments to day‐to‐day management (first order), a more substantial institutional redesign (second order), or a rethinking of the principles and values underpinning governance goals (third‐order or meta governance). Recognizing that there are limits to the extent to which governance systems can match the systems they are designed to govern, an understanding of governance challenges can also act as a reality check for potential interventions (Song & Chuenpagdee, 2010; Scholtens, 2015). For instance, the feasibility of genetic, ecological, and environmental interventions, identified to support coral reef conservation in a recent review (National Academies of Sciences Engineering & Medicine, 2019), should be considered in relation to context‐specific governance challenges. Governance goals can be amended in light of governability challenges to identify what can be realistically achieved rather than seeking success in relation to ideal images of governance. This requires an evaluation of prevalent images of how coral reefs should be governed, and the management tools associated with these, to assess their appropriateness to a particular context, rather than the acceptance of particular management approaches as “cure‐alls” (Ostrom et al., 2007).

Understanding the discourse of local and national governance actors is vital to improve coral reef governance and conservation outcomes. We examined commonalities and differences in the framing of the coral reef governance problem. National and local actors are involved in ongoing efforts to improve coral reef governance in order to effectively address local stressors, such as overfishing, pollution, and coastal development. Given the range of actors across different sectors and scales who interact in formal and informal ways, shared mental models (or at least an understanding of where mental models diverge) can be important in coming to agreement on appropriate solutions (Mahon et al., 2005; Jentoft, 2007; Song et al., 2013). Using an interactive governance approach to map respondents’ narratives of governance challenges against the different orders of governance, our findings highlighted an overall pattern weighted toward first‐ and second‐order governance and the (predominantly social) context of the system to be governed. Least attention was paid to third‐order issues, those of metagovernance. In the quest to improve governance, adding new policies and management approaches to existing flawed arrangements can make it increasingly difficult to challenge the status quo and achieve necessary governance transformations (Kelly et al., 2019). Interventions to improve first and second orders of governance may, therefore, not be effective without also paying attention to metagovernance. This is critical in the context of calls for transformative change in coral reef governance; radical action is required to improve governance at multiple scales to address key threats, such as climate change (Kennedy et al., 2013; Morrison et al., 2020a). Such transformations will require the engagement and support of actors across all scales to achieve equity and sustainability outcomes (Blythe et al., 2021), necessitating the engagement of practitioners and policy makers in wider conversations about governance. Though many improvements to governance may be incremental, a shared vision and goals can support a transformative agenda that such smaller changes contribute to (Patterson et al., 2017). Our findings point to a need to strengthen local appreciation of what is involved in governance, beyond making and enforcing rules, to consider deliberating values and principles, evaluating governability, and building appropriate multiscale capacity to steer reef governance in the right direction.

## Supporting information

Appendix S1. Interview guide
Appendix S2. Frequency of codes underpinning governance themes

## References

[cobi13933-bib-0001] Agardy, T. , di Sciara, G. N. , & Christie, P. (2011). Mind the gap: Addressing the shortcomings of marine protected areas through large scale marine spatial planning. Marine Policy, 35, 226–232.

[cobi13933-bib-0002] Agostini, V. N. , Margles, S. W. , Schill, S. R. , Knowles, J. E. , & Blyther, R. J. (2010). Marine zoning in Saint Kitts and Nevis: A path towards sustainable management of marine resources. The Nature Conservancy.

[cobi13933-bib-0003] Andrello, M. , Darling, E. S. , Wenger, A. , Suárez‐Castro, A. F. , Gelfand, S. , & Ahmadia, G. N. (2021). A global map of human pressures on tropical coral reefs. Conservation Letters, 15(1), e12858.

[cobi13933-bib-0004] Baird, J. , Plummer, R. , Schultz, L. , Armitage, D. , & Bodin, Ö. (2019). How does socio‐institutional diversity affect collaborative governance of social–ecological systems in practice? Environmental Management, 63, 200–214. 30426161 10.1007/s00267-018-1123-5

[cobi13933-bib-0005] Basurto, X. , Gelcich, S. , & Ostrom, E. (2013). The social–ecological system framework as a knowledge classificatory system for benthic small‐scale fisheries. Global Environmental Change, 23, 1366–1380.

[cobi13933-bib-0006] Bavinck, M. , Salagrama, V. , Bavinck, M. , & Salagrama, V. (2008). Assessing the governability of capture fisheries in the Bay of Bengal — A conceptual enquiry. Journal of Transdisciplinary Environmental Studies, 7, 1602–2297.

[cobi13933-bib-0007] Bennett, N. J. , & Satterfield, T. (2018). Environmental governance: A practical framework to guide design, evaluation, and analysis. Conservation Letters, 11, 1–13.

[cobi13933-bib-0008] Berkes, F. (2010). Linkages and multilevel systems for matching governance and ecology: Lessons from roving bandits. Bulletin of Marine Science, 86, 235–250.

[cobi13933-bib-0009] Blythe, J. , Cohen, P. , Eriksson, H. , Cinner, J. , Boso, D. , Schwarz, A. M. , & Andrew, N. (2017). Strengthening post‐hoc analysis of community‐based fisheries management through the social‐ecological systems framework. Marine Policy, 82, 50–58.

[cobi13933-bib-0010] Blythe, J. L. , Armitage, D. , Bennett, N. J. , Silver, J. J. , & Song, A. M. (2021). The politics of ocean governance transformations. Frontiers in Marine Science, 8, 634718.

[cobi13933-bib-0076] Box, S. , & Canty, S. (2010). The long and short term economic drivers of overexploitation in honduran coral reef fisheries. Pages 1‐9 Proceedings of the 63rd Gulf and Caribbean Fisheries Institute. November 1‐5, 2010. San Juan, Puerto Rico.

[cobi13933-bib-0011] Brewer, T. D. (2013). Dominant discourses, among fishers and middlemen, of the factors affecting coral reef fish distributions in Solomon Islands. Marine Policy, 37, 245–253.

[cobi13933-bib-0012] Bridgewater, P. , & Kim, R. E. (2021). The Ramsar Convention on Wetlands at 50. Nature Ecology and Evolution, 5, 268–270. 33526891 10.1038/s41559-021-01392-5

[cobi13933-bib-0013] Burke, L. , & Maidens, J. (2004). Reefs at risk in the Caribbean. Washington, DC: World Resources Institute.

[cobi13933-bib-0014] Burke, L. , Reytar, K. , Spalding, M. , & Perry, A. L. (2011). Reefs at risk revisited. Washington, DC: World Resources Institute.

[cobi13933-bib-0015] Cho, L. (2005). Marine protected areas: A tool for integrated coastal management in Belize. Ocean & Coastal Management, 48, 932–947.

[cobi13933-bib-0016] Christie, P. , & White, A. T. (2007). Best practices for improved governance of coral reef marine protected areas. Coral Reefs, 26, 1047–1056.

[cobi13933-bib-0017] Chuenpagdee, R. (2011). Interactive governance for marine conservation: An illustration. Bulletin of Marine Science, 87, 197–211.

[cobi13933-bib-0018] Chuenpagdee, R. , & Jentoft, S. (2007). Step zero for fisheries co‐management: What precedes implementation. Marine Policy, 31, 657–668.

[cobi13933-bib-0019] Chuenpagdee, R. , & Jentoft, S. (2009). Governability assessment for fisheries and coastal systems: A reality check. Human Ecology, 37, 109–120.

[cobi13933-bib-0020] Chuenpagdee, R. , Jentoft, S. , Bavinck, M. , & Kooiman, J. (2013a). Governability – New directions in fisheries governance. In M. Bavinck , R. Chuenpagdee , S. Jentoft , & J. Kooiman (Eds.), Governability of fisheries and aquaculture: Theory and applications (pp. 3–8). Amsterdam: MARE Publication Series 7, Springer.

[cobi13933-bib-0021] Chuenpagdee, R. , Pascual‐Fernández, J. J. , Szeliánszky, E. , Luis Alegret, J. , Fraga, J. , & Jentoft, S. (2013b). Marine protected areas: Re‐thinking their inception. Marine Policy, 39, 234–240.

[cobi13933-bib-0022] Cinner, J. E. , Zamborain‐Mason, J. , Gurney, G. G. , Graham, N. A. J. , MacNeil, M. A. , Hoey, A. S. , Mora, C. , Villéger, S. , Maire, E. , McClanahan, T. R. , Maina, J. M. , Kittinger, J. N. , Hicks, C. C. , D'agata, S. , Huchery, C. , Barnes, M. L. , Feary, D. A. , Williams, I. D. , Kulbicki, M. , …., Mouillot, D. (2020). Meeting fisheries, ecosystem function, and biodiversity goals in a human‐dominated world. Science, 368, 307–311. 32299952 10.1126/science.aax9412

[cobi13933-bib-0023] Cinner, J. E. , Basurto, X. , Fidelman, P. , Kuange, J. , Lahari, R. , & Mukminin, A. (2012). Institutional designs of customary fisheries management arrangements in Indonesia, Papua New Guinea, and Mexico. Marine Policy, 36, 278–285.

[cobi13933-bib-0074] CIA. (2013). The World Factbook 2013‐2014. Washington, DC: Central Intelligence Agency. https://www.cia.gov/library/publications/the-world-factbook/index.html

[cobi13933-bib-0075] CRFM. (2011). Report of Seventh Annual Scientific Meeting. Kingstown, St Vincent and the Grenadines. June 2011. Caribbean Regional Fisheries Mechanism Secretariat, https://clmeplus.org/app/uploads/2020/06/Supplement-1_Scientificmeetings2011.pdf

[cobi13933-bib-0024] Cox, M. , Arnold, G. , Tomás, S. , & Villamayor Tomas, S. (2010). A review of design principles for community‐based natural resource management. Ecology & Society, 15, 38.

[cobi13933-bib-0025] Dawson, N. , Martin, A. , & Danielsen, F. (2018). Assessing equity in protected area governance: Approaches to promote just and effective conservation. Conservation Letters, 11, e12388.

[cobi13933-bib-0026] Eddy, T. D. , Lam, V. W. Y. , Reygondeau, G. , Cisneros‐Montemayor, A. M. , Greer, K. , Palomares, M. L. D. , Bruno, J. F. , Ota, Y. , & Cheung, W. W. L. (2021). Global decline in capacity of coral reefs to provide ecosystem services. One Earth, 4, 1278–1285.

[cobi13933-bib-0027] Epstein, G. , Pittman, J. , Alexander, S. M. , Berdej, S. , Dyck, T. , Kreitmair, U. , Raithwell, K. J. , Villamayor‐Tomas, S. , Vogt, J. , & Armitage, D. (2015). Institutional fit and the sustainability of social‐ecological systems. Current Opinion in Environmental Sustainability, 14, 34–40.

[cobi13933-bib-0077] Engle, N. L. , & Lemos, M. C. (2010). Unpacking governance: Building adaptive capacity to climate change of river basins in Brazil. Global Environmental Change, 20, 4–13.

[cobi13933-bib-0028] Fanning, L. , Mahon, R. , McConney, P. , Angulo , J. , Burrows , F. , Chakalall, B. , Gil, D. , Haughton, M. , Heileman, S. , Martínez, S. , Ostine, L. , Adrian, O. , Scott, P. , Terrence, P. , Arroya, C. S. , Bertha, S. , & Cesar, T. (2007). A large marine ecosystem governance framework. Marine Policy, 31, 434–443.

[cobi13933-bib-0029] Fanning, L. , Mahon, R. , & Mcconney, P. (2013). Applying the large marine ecosystem (LME) governance framework in the Wider Caribbean Region. Marine Policy, 42, 99–110.

[cobi13933-bib-0030] Fanning, L. , Mahon, R. , & McConney, P. (2009). Focusing on living marine resource governance: The Caribbean Large Marine Ecosystem and Adjacent Areas Project. Coastal Management, 37, 219–234.

[cobi13933-bib-0031] Folke, C. , Pritchard, L. , Berkes, F. , Colding, J. , & Svedin, U. (2007). The problem of fit between ecosystems and institutions: Ten years later. Ecology and Society, 12, 30.

[cobi13933-bib-0032] Forster, J. , Turner, R. A. , Fitzsimmons, C. , Peterson, A. M. , Mahon, R. , & Stead, S. M. (2017). Evidence of a common understanding of proximate and distal drivers of reef health. Marine Policy, 84, 263–272.

[cobi13933-bib-0033] Fortnam, M. P. (2019). Forces opposing sustainability transformations: Institutionalization of ecosystem‐based approaches to fisheries management. Ecology and Society, 24, 33.

[cobi13933-bib-0034] Friedman, R. S. , Law, E. A. , Bennett, N. J. , Ives, C. D. , Thorn, J. P. R. , & Wilson, K. A. (2018). How just and just how? A systematic review of social equity in conservation research. Environmental Research Letters, 13, 053001.

[cobi13933-bib-0035] Gelcich, S. , Godoy, N. , & Castilla, J. C. (2009). Artisanal fishers’ perceptions regarding coastal co‐management policies in Chile and their potentials to scale‐up marine biodiversity conservation. Ocean & Coastal Management, 52, 424–432.

[cobi13933-bib-0036] Gill, D. A. , Mascia, M. B. , Ahmadia, G. N. , Glew, L. , Lester, S. E. , Barnes, M. , Craigie, I. , Darling, E. S. , Free, C. M , Geldmann, J. , Holst, S. , Jensen, O. P. , White, A. T. , Basurto, X. , Coad, L. , Gates, R. D. , Guannel, G. , Mumby, P. J. , Thomas, H. , …. Fox, H. E. (2017). Capacity shortfalls hinder the performance of marine protected areas globally. Nature, 543, 665–669. 28329771 10.1038/nature21708

[cobi13933-bib-0037] Gurney, G. G. , Mangubhai, S. , Fox, M. , Kiatkoski Kim, M. , & Agrawal, A. (2021). Equity in environmental governance: Perceived fairness of distributional justice principles in marine co‐management. Environmental Science and Policy, 124, 23–32.

[cobi13933-bib-0078] Harborne, A. R. , Mumby, P. J. , & Ferrari, R. (2011). The effectiveness of different meso‐scale rugosity metrics for predicting intra‐habitat variation in coral‐reef fish assemblages. Environmental Biology of Fishes, 94, 431–442.

[cobi13933-bib-0038] Hoegh‐Guldberg, O. , Poloczanska, E. S. , Skirving, W. , & Dove, S. (2007). Coral reefs under rapid climate change and ocean acidification. Science, 318, 1737–1742. 18079392 10.1126/science.1152509

[cobi13933-bib-0039] Hughes, T. P. , Kerry, J. T. , Álvarez‐Noriega, M. , Álvarez‐Romero, J. G. , Anderson, K. D. , Baird, A. H. , Babcock, R. C. , Beger, M. , Bellwood, D. R. , Berkelmans, R. , Bridge, T. C. , Butler, I. R. , Byrne, M. , Cantin, N. E. , Comeau, S. , Connolly, S. R. , Cumming, G. S. , Dalton, S. J. , Diaz‐Pulido, G. , …. Wilson, S. K. (2017a). Global warming and recurrent mass bleaching of corals. Nature, 543, 373–377. 28300113 10.1038/nature21707

[cobi13933-bib-0040] Hughes, T. P. , Barnes, M. L. , Bellwood, D. R. , Cinner, J. E. , Cumming, G. S. , Jackson, J. B. C. , Kleypas, J. , van de Leemput, I. A. , Lough, J. M. , Morrison, T. H. , Palumbi, S. R. , van Nes, E. H. , & Scheffer, M. (2017b). Coral reefs in the Anthropocene. Nature, 546, 82–90. 28569801 10.1038/nature22901

[cobi13933-bib-0041] Jackson, J. B. C. , Donovan, M. K. , Cramer, K. L. , & Lam, W. (2014). Status and trends of Caribbean coral reefs: 1970–2012. Global Coral Reef Monitoring Network. Gland, Switzerland: IUCN.

[cobi13933-bib-0042] Jentoft, S. (2007). Limits of governability: Institutional implications for fisheries and coastal governance. Marine Policy, 31, 360–370.

[cobi13933-bib-0043] Jentoft, S. , van Son, T. C. , & Bjørkan, M. (2007). Marine protected areas: A governance system analysis. Human Ecology, 35, 611–622.

[cobi13933-bib-0079] Kaufmann, D. , Kraay, A. , & Zoido‐Lobatón, P. (2000). Governance matters from measurement to action. Finance & Development, 10–13.

[cobi13933-bib-0044] Kelly, C. , Ellis, G. , & Flannery, W. (2019). Unravelling persistent problems to transformative marine governance. Frontiers in Marine Science, 6, 213.

[cobi13933-bib-0045] Kennedy, E. V. , Iglesias‐Prieto, R. , Schönberg, C. H. L. , Wisshak, M. , Form, A. U. , Carricart‐Ganivet, J. P. , Fine, M. , Eakin, C. M. , & Mumby, P. J. (2013). Avoiding coral reef functional collapse requires local and global action. Current Biology, 23, 912–918. 23664976 10.1016/j.cub.2013.04.020

[cobi13933-bib-0046] Kooiman, J. , Bavinck, M. , Chuenpagdee, R. , Mahon, R. , & Pullin, R. (2008). Interactive governance and governability: An introduction. Journal of Transdisciplinary Environmental Studies, 7, 1–11.

[cobi13933-bib-0047] Kooiman, J. , Bavinck, M. , Jentoft, S. , & Pullin, R. (2005). Fish for life: Interactive governance for fisheries. Amsterdam: Amsterdam University Press.

[cobi13933-bib-0048] Mahon, R. (2008). Assessing governability of fisheries using the interactive governance approach: Preliminary examples from the Caribbean. Journal of Transdisciplinary Environmental Studies, 7(1), 1–12.

[cobi13933-bib-0049] Mahon, R. , Bavinck, M. , & Roy, R. N. (2005). Governance in action. In J. Kooiman , M. Bavinck , S. Jentoft , & R. Pullin (Eds.), Fish for life: Interactive governance for fisheries 2 (pp. 353–376). Amsterdam: Amsterdam University Press.

[cobi13933-bib-0050] Mahon, R. , Fanning, L. , & McConney, P. (2014). Assessing and facilitating emerging regional ocean governance arrangements in the Wider Caribbean Region. Ocean Yearbook, 28, 631–671.

[cobi13933-bib-0080] McConney, P. , Mahon, R. , & Oxenford, HA. (2003). Barbados case study?: The fisheries advisory committee. Caribbean Coastal Co‐management Guidelines Project. Caribbean Conservation Association, Barbados.

[cobi13933-bib-0051] McConney, P. , Mahon, R. , & Pomeroy, R. (2007). Challenges facing coastal resource co‐management in the Caribbean. In D. R. Armitage , F. Berkes , & N. C. Doubleday (Eds.), Adaptive co‐management: Collaboration, learning and multi‐level governance (pp. 105–124). Vancouver, BC: UBC Press.

[cobi13933-bib-0052] McConney, P. , & Pena, M. (2012). Capacity for (co)management of marine protected areas in the Caribbean. Coastal Management, 40, 268–278.

[cobi13933-bib-0053] Mora, C. (2008). A clear human footprint in the coral reefs of the Caribbean. Proceedings of the Royal Society B, 275, 767–773. 18182370 10.1098/rspb.2007.1472 PMC2596901

[cobi13933-bib-0054] Morrison, T. H. , Adger, W. N. , Brown, K. , Lemos, M. C. , Huitema, D. , Phelps, J. , Evans, L. , Cohen, P. , Song, A. M. , Turner, R. , Quinn, T. , & Hughes, T. P. (2019a). The black box of power in polycentric environmental governance. Global Environmental Change, 57, 101934.

[cobi13933-bib-0055] Morrison, T. H. , Hughes, T. P. , Adger, W. N. , Brown, K. , Barnett, J. , & Lemos, M. C. (2019b). Save reefs to rescue all ecosystems. Nature, 573, 333–336. 31534250 10.1038/d41586-019-02737-8

[cobi13933-bib-0056] Morrison, T. H. , Adger, N. , Barnett, J. , Brown, K. , Possingham, H. , & Hughes, T. (2020a). Advancing coral reef governance into the Anthropocene. One Earth, 2, 64–74.

[cobi13933-bib-0057] Morrison, T. H. , Adger, W. N. , Brown, K. , Hettiarachchi, M. , Huchery, C. , Lemos, M. C. , & Hughes, T. P. (2020b). Political dynamics and governance of World Heritage ecosystems. Nature Sustainability, 3, 947–955.

[cobi13933-bib-0058] Mumby, P. J. , & Steneck, R. S. (2008). Coral reef management and conservation in light of rapidly evolving ecological paradigms. Trends in Ecology & Evolution, 23, 555–63. 18722687 10.1016/j.tree.2008.06.011

[cobi13933-bib-0059] National Academies of Sciences Engineering and Medicine . (2019). A research review of interventions to increase the persistence and resilience of coral reefs. Washington, DC: National Academies Press.

[cobi13933-bib-0060] Ostrom, E. (2007). A diagnostic approach for going beyond panaceas. Proceedings of the National Academy of Sciences of the United States of America, 104, 15181–15187. 17881578 10.1073/pnas.0702288104 PMC2000497

[cobi13933-bib-0061] Ostrom, E. (2009). A general framework for analyzing sustainability of social‐ecological systems. Science, 325, 419–422. 19628857 10.1126/science.1172133

[cobi13933-bib-0062] Ostrom, E. , Janssen, M. A. , & Anderies, J. M. (2007). Going beyond panaceas. Proceedings of the National Academy of Sciences of the United States of America, 104, 15176–15178. 17881583 10.1073/pnas.0701886104 PMC2000490

[cobi13933-bib-0063] Patterson, J. , Schulz, K. , Vervoort, J. , Van Der, H. S. , Widerberg, O. , Adler, C. , Hurlbert, M. , Anderton, K. , Sethi, M. , & Barau, A. (2017). Exploring the governance and politics of transformations towards sustainability. Environmental Innovation and Societal Transitions, 24, 1–16.

[cobi13933-bib-0064] Sale, P. F. (2008). Management of coral reefs: Where we have gone wrong and what we can do about it. Marine Pollution Bulletin, 56, 805–809. 18456286 10.1016/j.marpolbul.2008.04.009

[cobi13933-bib-0065] Scholtens, J. (2015). Limits to the governability of transboundary fisheries: Implications for small‐scale fishers in Northern Sri Lanka and beyond. In S. Jentoft & R. Chuenpagdee (Eds.), Interactive governance for small‐scale fisheries. MARE Publication Series 13, Springer International, p. 515–538.

[cobi13933-bib-0066] Song, A. M. , & Chuenpagdee, R. (2010). Operationalizing governability: A case study of a Lake Malawi fishery. Fish and Fisheries, 11, 235–249.

[cobi13933-bib-0067] Song, A. M. , Chuenpagdee, R. , & Jentoft, S. (2013). Values, images, and principles: What they represent and how they may improve fisheries governance. Marine Policy, 40, 167–175.

[cobi13933-bib-0068] Turner, R. A. , Fitzsimmons, C. , Forster, J. , Mahon, R. , Peterson, A. , & Stead, S. M. (2014). Measuring good governance for complex ecosystems: Perceptions of coral reef‐dependent communities in the Caribbean. Global Environmental Change, 29, 105–117.

[cobi13933-bib-0069] Turner, R. A. , Forster, J. , Fitzsimmons, C. , Gill, D. , Mahon, R. , Peterson, A. , & Stead, S. (2017). Social fit of coral reef governance varies among individuals. Conservation Letters, 11(3), e12422.

[cobi13933-bib-0070] Wilkinson, C. , & Souter, D. (2008). Status of Caribbean coral reefs after bleaching and hurricanes in 2005. Townsville: Global Coral Reef Monitoring Network, and Reef and Rainforest Research Centre.

[cobi13933-bib-0071] Ziegler, J. P. , Jones, S. E. , & Solomon, C. T. (2019). Local stakeholders understand recreational fisheries as social‐ecological systems but do not view governance systems as influential for system dynamics. International Journal of the Commons, 13, 1035–1048.

