# Supplementary material for: Expanding narratives of governance constraints to improve coral reef conservation
Source: Conserv Biol. 2022 Jul 11;36(5):e13933. doi: 10.1111/cobi.13933 (PMC9795921; doi:10.1111/cobi.13933)
Supplement: Supplementary file 1 — Appendix S1. Interview guide Appendix S2. Frequency of codes underpinning governance themes [file COBI-36-0-s001.docx]

Appendix S1. Interview guide

***Background***

Name

Current position

Organisation/department

What are the main purposes and priorities of your organisation or department?

What are your main responsibilities?

Do you have any other relevant experience or responsibilities related to reefs?

Approximately how many members/employees are there? (Who? How do they become involved?)

When was your organization created? (And who was responsible for its creation?)

What is the main source of funding for the organisation?

***Perception of status of reefs and impacts to reefs***

What are the most important types of reef resource use in this area/country?

What attributes of the reef do these resource users depend on?

How healthy do you think the reefs are here/in your country, from 1 to 4, where 1 is very unhealthy and 4 is very healthy?

What do you think are the most important impacts to reefs in your area/country?

What are the causes of these impacts?

Have you noticed or heard about any changes in coral reefs over the past 10years?

Have you noticed or heard of any other changes in the marine environment over the past 10 years?

What do you think will be the main impacts of climate change on the health of reefs in this area/country?

***Reef management***

Which of the following reef management measures (tools) are in place in this area/country?

To what extent do they have an impact on reef health and why? (Rate impact where 1 = negative, 2 = no effect, 3 = uncertain, 4 = likely positive, 5 = proven positive)

- Technical measures e.g. mooring buoys and fisheries technical measures
- Temporal measures e.g. seasonal closures
- Spatial measures e.g. MPAs
- Ecological monitoring and research
- Social surveys and research
- Alternative livelihoods and economic incentives
- Management approaches e.g. ecosystem-based and co-management
- Environmental education
- Communication and participatory processes e.g. workshops and forums, and stakeholder involvement
- Manuals and guidelines for reef managers
- Software support and decision-making tools e.g. computer mapping and modelling
- Legislation and legal instruments e.g. fisheries or pollution laws
- Policy and management plans

What do you think about current management of reefs in the area/country?

Are there mechanisms in place to enforce these management measures? What are they?

What do you think about the enforcement mechanisms? (e.g. fairness)

Are there any informal rules or community arrangements about how people use reef resources? If so, what are they and how do you think they affect reef health?

What could be done in the area/country to improve the health of coral reefs?

***Decision-making***

Could you describe how decisions about reef management are taken in the area/country? How, and by whom?

Are there any forums or meetings for departments, organisations or stakeholders from different sectors to discuss issues related to reefs?

Do you think local management priorities for reefs are the same as at the national level?

Do you think decisions about reef management are made at the right organizational level?

How flexible are current management mechanisms? E.g. if the reef status changes, could reef management change accordingly? How would changing reef management be achieved?

In terms of reef management, is there one thing you would like to do but can’t? What are the challenges?

Thinking more generally, do you perceive any challenges to managing reefs effectively?

***Stakeholder involvement***

Is there an opportunity for stakeholders to be involved in reef management? If so, who and how?

Have any decisions about reef management disadvantaged any reef users? If so who and how?

If decisions are made about the management of reefs, is information provided to stakeholders to explain why a particular decision was taken?

Are there ways people can challenge the rules, laws or decisions made regarding reef management?

The previous questions about management, cooperation, decision-making etc, are all about reef governance. Do you have any suggestions for how governance could be improved?

***National situation***

What are the main priorities for the government?

How important are reef management issues in comparison to other government priorities?

Appendix S2. Frequency of codes underpinning governance themes

| Theme | Codes  Perceived challenges related to: | Frequency mentioned (%) | | | | |
| --- | --- | --- | --- | --- | --- | --- |
|  |  | BBD (14) | SKN (25) | BZE (38) | HON (33) | Total (110) |
| Socio-economic context | Education & awareness | 71 | 60 | 47 | 48 | 55 |
|  | Socio-economic pressures (general) | 50 | 40 | 42 | 70 | 52 |
|  | Diversity & conflict | 64 | 32 | 18 | 21 | 28 |
|  | Livelihood dependency | 14 | 28 | 26 | 36 | 28 |
|  | Alternative livelihoods | 0 | 16 | 21 | 39 | 23 |
|  | Tradition & culture | 36 | 24 | 3 | 12 | 15 |
|  | Economic downturn | 7 | 12 | 16 | 6 | 11 |
|  | Sewage system | 29 | 0 | 3 | 18 | 10 |
|  | Small society | 0 | 20 | 11 | 3 | 9 |
|  | Fishing as a safety net | 0 | 20 | 8 | 6 | 9 |
|  | Immigration | 0 | 4 | 8 | 15 | 8 |
|  | Geography & location | 0 | 0 | 16 | 6 | 7 |
|  | Population increase | 0 | 4 | 5 | 15 | 7 |
|  | Poverty | 0 | 0 | 5 | 15 | 6 |
|  | Past experiences | 7 | 0 | 5 | 12 | 6 |
|  | External impacts | 0 | 0 | 13 | 0 | 5 |
|  | Market demand | 0 | 4 | 5 | 6 | 5 |
|  | Unemployment | 0 | 0 | 3 | 9 | 4 |
|  | Education systems | 0 | 8 | 3 | 3 | 4 |
|  | Political differences | 0 | 12 | 0 | 0 | 3 |
|  | Sugar industry decline | 0 | 12 | 0 | 0 | 3 |
|  | Political unrest | 0 | 0 | 0 | 6 | 2 |
|  | Drug problems | 0 | 0 | 0 | 6 | 2 |
|  | Crime | 0 | 0 | 0 | 6 | 2 |
|  | Geopolitical complexity | 7 | 0 | 0 | 0 | 1 |
|  | Social differences | 0 | 4 | 0 | 0 | 1 |
|  | Poor health | 0 | 0 | 0 | 3 | 1 |
|  | Access to seafood | 0 | 0 | 3 | 0 | 1 |
|  | Historical legacy | 7 | 0 | 0 | 0 | 1 |
| Ecological context | Scale (general) | 57 | 40 | 74 | 30 | 52 |
|  | Ecological connectivity | 43 | 28 | 21 | 15 | 24 |
|  | Scale of climate change | 21 | 8 | 29 | 3 | 16 |
|  | Ecological range | 0 | 4 | 21 | 9 | 11 |
|  | Complex system dynamics | 21 | 4 | 3 | 18 | 11 |
|  | Temporal dynamics | 0 | 4 | 3 | 3 | 4 |
|  | Hurricanes | 0 | 4 | 8 | 0 | 4 |
| Management inputs | Resources & capacity | 71 | 88 | 89 | 91 | 88 |
|  | Dependence on donor funds | 0 | 0 | 5 | 18 | 7 |
|  | Changing funding climate | 0 | 0 | 3 | 0 | 1 |
| Management processes | Slow processes | 21 | 28 | 18 | 36 | 26 |
|  | Ad hoc management | 14 | 20 | 13 | 3 | 12 |
|  | Misuse of funds | 0 | 0 | 5 | 3 | 3 |
|  | Ineffective use of resources & capacity | 0 | 0 | 3 | 3 | 2 |
|  | Reactive management | 7 | 0 | 0 | 0 | 1 |
| Management outputs | Ineffective management (general) | 93 | 100 | 95 | 94 | 96 |
|  | Enforcement | 86 | 96 | 95 | 88 | 93 |
|  | Implementation | 50 | 60 | 42 | 52 | 51 |
|  | Education & awareness programs | 43 | 60 | 47 | 42 | 49 |
|  | Need for marine protected areas | 0 | 64 | 32 | 27 | 34 |
|  | Monitoring & inspection | 0 | 60 | 24 | 18 | 28 |
|  | Management plans | 14 | 12 | 24 | 36 | 24 |
|  | Moorings | 7 | 24 | 18 | 6 | 15 |
|  | Seasonal closures | 0 | 28 | 11 | 0 | 10 |
|  | Recycling & waste collection | 7 | 4 | 5 | 9 | 6 |
|  | Artificial reefs & reef restoration | 0 | 8 | 5 | 6 | 5 |
|  | Watershed management | 14 | 0 | 0 | 9 | 5 |
|  | Incentives | 0 | 0 | 8 | 3 | 4 |
|  | Reliance on marine protected areas | 0 | 0 | 3 | 0 | 1 |
|  | Compensation | 0 | 0 | 0 | 0 | 1 |
| Institutional structures | Appropriate institutional structures (general) | 57 | 48 | 53 | 48 | 52 |
|  | Clarity of roles & responsibilities | 21 | 24 | 16 | 27 | 22 |
|  | Appropriate fit to scale | 7 | 16 | 11 | 18 | 14 |
|  | Specific marine management agency | 36 | 8 | 5 | 0 | 8 |
|  | Bureaucracy | 0 | 0 | 5 | 6 | 4 |
| Leadership and power | Prioritisation | 86 | 84 | 61 | 82 | 76 |
|  | Political will | 50 | 48 | 29 | 48 | 43 |
|  | Conflict of interest | 21 | 28 | 24 | 30 | 26 |
|  | Ongoing commitment | 21 | 16 | 16 | 42 | 25 |
|  | Willingness & capacity | 21 | 16 | 8 | 18 | 15 |
|  | Corruption | 7 | 8 | 21 | 18 | 15 |
|  | Susceptibility to political change | 14 | 8 | 8 | 24 | 14 |
|  | Insufficient authority | 0 | 12 | 5 | 24 | 12 |
|  | Leadership | 14 | 8 | 0 | 9 | 6 |
|  | Political influence | 14 | 4 | 3 | 6 | 5 |
|  | Political reprisals | 7 | 4 | 0 | 0 | 2 |
|  | Public pressure | 0 | 0 | 0 | 3 | 1 |
|  | Willingness to confront complex issues | 0 | 0 | 0 | 3 | 1 |
| Legislation and regulations | Weak policy, legislation or regulations (general) | 71 | 84 | 61 | 61 | 68 |
|  | Reliance on informal governance | 29 | 20 | 16 | 21 | 20 |
|  | Inadequate penalties | 7 | 4 | 13 | 15 | 12 |
|  | Outdated laws | 7 | 4 | 8 | 15 | 9 |
|  | Tenure systems | 7 | 0 | 8 | 3 | 5 |
|  | Mechanisms for change | 7 | 0 | 0 | 0 | 1 |
| Engagement and participation | Engagement (general) | 79 | 68 | 74 | 76 | 75 |
|  | Stakeholder support | 43 | 48 | 53 | 55 | 52 |
|  | Stewardship | 0 | 20 | 11 | 12 | 13 |
|  | Little public demand/pressure | 21 | 12 | 11 | 0 | 10 |
|  | Collective action | 14 | 8 | 5 | 12 | 9 |
|  | Stakeholder voice | 14 | 4 | 3 | 3 | 5 |
|  | Stakeholder fatigue | 0 | 4 | 3 | 3 | 3 |
|  | Weak stakeholder engagement | 7 | 0 | 5 | 0 | 3 |
|  | Media coverage | 0 | 0 | 5 | 0 | 3 |
| Research and information | Information & research | 71 | 60 | 45 | 58 | 55 |
|  | Attention to science & technical advice | 14 | 16 | 16 | 18 | 17 |
|  | Systems understanding | 7 | 12 | 11 | 12 | 11 |
|  | Dissemination | 0 | 4 | 8 | 6 | 5 |
|  | Examples & best practices | 0 | 0 | 3 | 6 | 3 |
|  | Coordination of research | 0 | 0 | 5 | 0 | 2 |
| Connectivity | Cooperation & integration (general) | 64 | 56 | 74 | 82 | 72 |
|  | Formal cooperation mechanisms | 14 | 12 | 18 | 12 | 15 |
|  | Incomplete policy cycles | 7 | 8 | 13 | 12 | 11 |
| Meta-governance | Economic case for conservation | 7 | 20 | 16 | 15 | 15 |
|  | Shared vision | 14 | 16 | 8 | 12 | 12 |
|  | Clarity of goals | 14 | 12 | 5 | 12 | 10 |
|  | Values | 21 | 4 | 8 | 6 | 9 |
|  | Short-term outlook | 29 | 16 | 0 | 6 | 9 |
| Quality of process | Fairness | 36 | 20 | 42 | 39 | 35 |
|  | Transparency | 43 | 4 | 26 | 30 | 25 |
|  | Legitimacy | 7 | 20 | 16 | 36 | 22 |
|  | Trust | 14 | 0 | 21 | 21 | 15 |
|  | Flexibility | 14 | 24 | 13 | 12 | 15 |
|  | Accountability | 14 | 8 | 8 | 12 | 11 |
|  | Credibility | 0 | 0 | 3 | 3 | 2 |
